# Vaccination

**Published:** 1860-05

**Authors:** 


### Vaccination.

We have ascertained that vaccine is difficult to get, in fact scarcely procurable in this city at the present time, and for some time past. This is a most dangerous state of affairs, particularly when we consider that small pox was prevalent last summer, and that it has continued more or less ever since.

When the immortal Jenner discovered vaccine and fully proved its efficacy, the world rose to applaud the greatest benefactor of his race, and mankind, emancipated from the scourge of a fearful plague, were everywhere alive to the vital importance of the antidote.

But as time rolls on, and the immunity from the pest caused by general vaccination, has lasted for years, a supineness on the subject and neglect of its momentous and imperative duties, seems to be creeping through at least our community.

Should this be so? Is the loathsome disease less fearful, or its ravages less deadly, on the unvaccinated now than formerly? The mournful answer is—decidedly not.

The practitioner can now behold in his rounds the same frightful phenomena which characterized the small pox when it decimated the human race. It is but a smouldering fire of pestilence, lurking and burrowing its way through the inflammable material of our immense and crowded population, and requires but neglect of vaccination and a hot summer's sun to fan it into a plague; yet on this mine of pestilence and death our population tread in fancied security, actively, almost morbidly, awake to the danger of yellow fever, but oblivious of another monster, which is in our midst, and which, if the yellow fever has "slain its thousands," has "slain its tens of thousands." Is not this a fearful state of things? yet such is the case.

What is the cause?—and where is the remedy? An all-engrossing interest in the spoils of office, and supreme indifference to the safety of the people on the part of our city officials, and a fancied security on the part of the multitude, because vaccination is so easy, and they suppose so attainable, is the cause. The remedy consists in having an immediate, a general vaccination, one which will be *compulsory* and *admit* of *no exceptions.*

That this is the *only* way, is proved by the past, for before the discovery of vaccination, every precaution which men's ingenuity could devise was tried, to ward off the pest, and found ineffectual.

Arising from the torrid swamps of Southern Arabia, and the hot, pestilential jungles of Hindostan, this dread messenger of death swept like a simoon over the earth, withering and destroying as it passed. Cities took the greatest precautions, and enacted the most stringent health measures, but in vain. In a few short weeks from the day the first was plague-stricken, the burning sun glared down on silent dwellings, and the rumbling of the death-carts alone woke the echo of the silent streets—*the population had gathered at the lazar-house.*

This awful necrology has ceased, because vaccination—the mighty antidote—has been discovered. However, small pox has *not* ceased, it is the *same* as ever. Let us, then, be wise in time. As well might we attempt to stem a torrent with a straw as stay the fearful pest, even by vaccination, *when* it is fully on us. Now, therefore, is the time; let us this spring have a general vaccination. Heaven knows we shall have enough of disease in the way of cholera infantum, &c., arising from filthy streets and crowded tenements, to contend with next summer, without adding thereto the most loathsome and deadly of all plagues.—*N. York Med. Press.*

